# Integrating sugarcane molasses into sequential cellulosic biofuel production based on SSF process of high solid loading

**DOI:** 10.1186/s13068-018-1328-0

**Published:** 2018-12-18

**Authors:** Meishan Fan, Shuaishuai Zhang, Guangying Ye, Hongdan Zhang, Jun Xie

**Affiliations:** College of Forestry and Landscape Architecture, Guangdong Engineering Technology Research Center of Agricultural and Forestry Biomass, Key Laboratory of Energy Plants Resource and Utilization, Ministry of Agriculture, South China Agricultural University, Guangzhou, 510642 People’s Republic of China

**Keywords:** Bioethanol, Biogas, Co-fermentation, High solid loading, Sugarcane bagasse, Sugarcane molasses

## Abstract

**Background:**

Sugarcane bagasse (SCB) is one of the most promising lignocellulosic biomasses for use in the production of biofuels. However, bioethanol production from pure SCB fermentation is still limited by its high process cost and low fermentation efficiency. Sugarcane molasses, as a carbohydrate-rich biomass, can provide fermentable sugars for ethanol production. Herein, to reduce high processing costs, molasses was integrated into lignocellulosic ethanol production in batch modes to improve the fermentation system and to boost the final ethanol concentration and yield.

**Results:**

The co-fermentation of pretreated SCB and molasses at ratios of 3:1 (mixture A) and 1:1 (mixture B) were conducted at solid loadings of 12% to 32%, and the fermentation of pretreated SCB alone at the same solid loading was also compared. At a solid loading of 32%, the ethanol concentrations of 64.10 g/L, 74.69 g/L, and 75.64 g/L were obtained from pure SCB, mixture A, and mixture B, respectively. To further boost the ethanol concentration, the fermentation of mixture B (1:1), with higher solid loading from 36 to 48%, was also implemented. The highest ethanol concentration of 94.20 g/L was generated at a high solid loading of 44%, with an ethanol yield of 72.37%. In addition, after evaporation, the wastewater could be converted to biogas by anaerobic digestion. The final methane production of 312.14 mL/g volatile solids (VS) was obtained, and the final chemical oxygen demand removal and VS degradation efficiency was 85.9% and 95.9%, respectively.

**Conclusions:**

Molasses could provide a good environment for the growth of yeast and inoculum. Integrating sugarcane molasses into sequential cellulosic biofuel production could improve the utilization of biomass resources.

## Background

Bioethanol, as a replacement and additive of gasoline, is a remarkable type of biofuel. Lignocellulosic biomass is regarded as a potential material for the production of cellulosic ethanol due to its relatively low cost and because it prevents the diversion of food crops to the production of bioethanol [1]. However, some disadvantages have limited the development of bioethanol production from lignocellulosic biomass, such as high enzyme costs and lower ethanol titers and yields [2, 3]. Currently, several methods have been proposed for the integration of grains or sugar juice to enhance the initial sugar concentration and improve the subsequent ethanol production [4]. Xu et al. [5] investigated the influence of starchy substrate addition on cellulosic ethanol production, demonstrating the improvement of the final ethanol concentration from 6.9 to 18.1 g/L. Ye et al. [6] obtained a final ethanol concentration of 82.83 g/L by the co-fermentation of sugarcane bagasse and *Dioscorea composita* hemls. Erdei et al. [7] achieved 99% of the theoretical ethanol yield using the mixtures of wheat straw and wheat meal. Damay et al. [8] found that mixing first-generation sugars and second-generation sugars could eliminate the effect of pretreatment inhibitors in the fermentation process to obtain ethanol yields of greater than 90%. Though significant advances have been made in improving ethanol production, a comprehensive understanding of the co-fermentation of sugarcane bagasse (SCB) and sugarcane molasses at different solid loadings remains unclear.

Sugarcane is the most important sugar crop in the tropical regions of the world, and it is widely planted in southern China [6]. SCB and molasses are both by-products of the cane sugar production. In China, approximately 36 million tons of sugarcane bagasse and 4 million tons of molasses are produced every year during sugar processing [9]. SCB, as the fibrous substance produced after juice extraction from sugarcane, has the characteristics of avoiding feedstock handling, being easy to collect and can be used directly in the sugar mill (i.e., no transportation costs) [10]. Due to the high cellulose contents of SCB, it is an attractive substrate for ethanol production. Molasses, containing a high concentration of fermentable sugars, is the noncrystallizable residue left after sugar crystallization from cane juice. It is usually used in alcohol distilleries without the steps of pretreatment and saccharification [11, 12]. Since SCB and molasses are produced at the same time in sugar production, co-fermentation of SCB and molasses as complementary materials would achieve the comprehensive economic benefits of sugarcane/sugar industry and gain higher resource utilization.

Due to the intact structure of sugarcane bagasse, pretreatment is conducted to effectively remove lignin and hemicellulose, disrupt the cellulose crystallinity, and increase the porosity of the biomass [13, 14]. Recently, various pretreatments have been proposed to separate the lignin and hemicelluloses from the lignocellulosic biomass, such as using acid, alkaline, steam explosion, wet oxidation, and organosolv techniques [15]. Compared with the other pretreatment methods, alkali pretreatment is more favorable because of its simple device, convenient operation, low cost, and high efficiency [13]. After alkali pretreatment, the contents of glucan and xylan in SCB were found to be increased due to delignification, making cellulose and hemicellulose available for the enzymatic degradation and resulting in an increase of sugar yield during the enzymatic saccharification [16]. Mcintosh et al. [17] observed a high rate of enzymatic saccharification and a high ethanol yield when using dilute alkaline pretreated wheat and sorghum straw as substrates.

Simultaneous saccharification and fermentation (SSF) is able to rapidly convert sugars into ethanol as soon as the saccharification of lignocellulosic biomass begins, which would relieve the inhibitory effect of high sugar contents on saccharomycetes [18]. Moreover, SSF has been extensively applied, because it can reduce the cost of equipment capital and the risk of contamination, as well as shorten the processing time compared with separate hydrolysis and fermentation (SHF) [19].

In general, the amount of biomass loadings is increased to meet a minimum economical ethanol distillation of 40 g/L [3]. A high biomass loading could enhance fermentable sugars and reduce the economic cost of ethanol distillation. However, it was shown that the ethanol yield decreased when the biomass loading was greater than 15% (w/w) [20]. In addition, high solid loadings mean that there is a large amount of biomass for fermentation, which increases the mixing difficulty and power consumption of stirred tank reactors [21, 22]. Furthermore, the concentration of byproducts (such as glycerol, lactic acid, and acetic acid) increases as the increment of biomass loading. These inhibitors have a negative impact on the fermenting microorganism [23]. It remains a challenge for the cellulosic ethanol production to achieve a high final ethanol concentration due to the difficulty of applying a high solid loading process in SSF in batch-mode reactions [24]. Hence, co-fermentation of mixed substrates would be a promising approach that could increase fermentable sugar concentrations and consequently result in a high final ethanol titer [25].

After the fermentation, the ethanol is collected and the residues in the stillage can be incinerated to produce steam or converted into animal feed. However, these methods cannot efficiently meet the requirements of sustainable manner [26]. It has been reported that stillage production is 10–20 times more than the volume of ethanol production [27]. Therefore, it is necessary to develop an environmentally friendly method for the utilization of stillage waste. Recently, anaerobic digestion has been recognized as an effective and feasible method to economically use the stillage [28]. The biogas produced from ethanol fermentation residues could be converted to another type of biofuel, which could provide renewable energy and reduce environmental pollution problems [29, 30].

In this research, compared with only pretreated SCB fermentation, two ratios of alkali-pretreated SCB and molasses were used to evaluate their influence on ethanol concentrations and yields during the SSF process. Furthermore, higher solid loadings of mixtures of pretreated SCB and molasses, with a ratio of 1:1, were investigated to help increase the ethanol concentration and to obtain a suitable conversion rate. In addition, the stillage was sequentially digested for biogas production to achieve a comprehensive biomass utilization.

## Results and discussion

### Chemical analysis of materials

As shown in Table 1, the molasses contained 9% of fructose, 6% of glucose, and 23% of sucrose (w/w). The chemical composition of raw SCB used in this experiment contained 38.43% cellulose, 24.30% xylan, 25.98% Klason lignin, and 6.14% acid soluble lignin (ASL). After alkali pretreatment, the composition of SCB was significantly altered, with a solid yield of 82.2%. The loss of solids was ascribed to the degradation of lignin. Owing to the delignification, the content of cellulose and xylan increased to 53.01% and 29.36%, respectively. The Klason lignin and ASL contents were decreased to 10.05% and 4.01%, respectively, which was in accordance with the results of decreased solid yields. Delignification provided a greater cellulose content of substrates available for enzymatic hydrolysis and improved the efficiency at high solid loadings [6].

**Table 1:** Chemical composition of the raw and pretreated SCB

Materials	Cellulose %^db^	Xylan %^db^	Klason lignin %^db^	ASL %^db^	Fructose %^wb^	Glucose %^wb^	Sucrose %^wb^	Solid yield %
Molasses					9 ± 0.4	6 ± 0.2	23 ± 0.7	30.61 ± 0.1^wb^
Untreated SCB	38.43 ± 0.50	24.3 ± 0.22	25.98 ± 0.14	6.14 ± 0.41				
Pretreated SCB	53.01 ± 0.70	29.36 ± 0.30	10.05 ± 0.05	4.01 ± 0.03				84.1 ± 0.5^db^

*ASL* acid soluble lignin

^db^: means dry basis

^wb^: means wet basis

### The fermentation of pure pretreated SCB

**Fig. 1:**
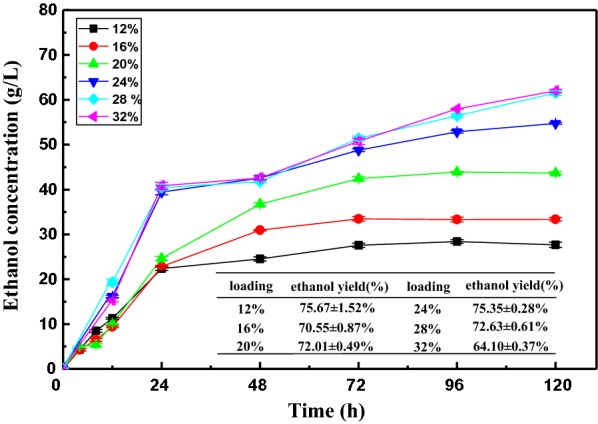
Ethanol concentrations and yields of SCB at different solid loadings in SSF

It is known to us that SCB is an excellent lignocellulosic biomass for ethanol production thanks to its relatively low lignin content and high cellulose content when using appropriate pretreatments [31]. Figure 1 depicts the ethanol concentration under different solid loadings, from 12 to 32% (w/v), as the fermentation time was extended during SSF. As shown, the ethanol concentration rapidly increased in the first 24 h owing to the high saccharification of the lignocellulosic biomass [32]. For the low loading of 12% to 20%, the concentration rose slightly and stabilized after 72 h. However, for the high loading of 24% to 32%, as the hydrolysis time was extended from 24 to 48 h, a very slow fermentation rate of less than 0.2 g/L/h was observed. This phenomenon could be due to the limitations of mass transfer or inhibition from the accumulated byproducts at high solid loadings [33]. Though the fermentation rate was low, the ethanol concentration increased gradually as the fermentation time was prolonged. The final ethanol concentration reached 27.69 g/L, 33.37 g/L, 43.67 g/L, 54.76 g/L, 61.52 g/L, and 62.01 g/L, as the solid loading increased from 12 to 32%. The maximum ethanol concentration (62.01 g/L) was achieved at a loading of 32%, with an ethanol yield of 64.1%, whereas a higher ethanol yield of 75.67% was obtained at a loading of 12%. These results suggest that fermentable yields decreased with the increase of biomass loading.

All ethanol concentrations of high-gravity fermentation reached the commercial production titers (> 40 g/L) except for the loadings of 12% and 16%. However, the saccharification and fermentation of lignocellulosic biomass required a longer time (120 h), and the liquidity of the reactant was limited by high substrate loadings, which increased the cost and reduced the economic efficiency [34]. Hence, it is unreasonable to generate higher ethanol concentrations by increasing only the solid loadings of pretreated SCB. Meanwhile, the fermentation of pretreated SCB alone would not be beneficial for yeast growth due to the lack of necessary nutrients. Hence, it is necessary to integrate substrates containing nutrient contents and high concentrations of sugars into the SCB ethanol fermentation process, thereby improving the ethanol concentration and yield [5].

### The co-fermentation of pretreated SCB and molasses

**Fig. 2:**
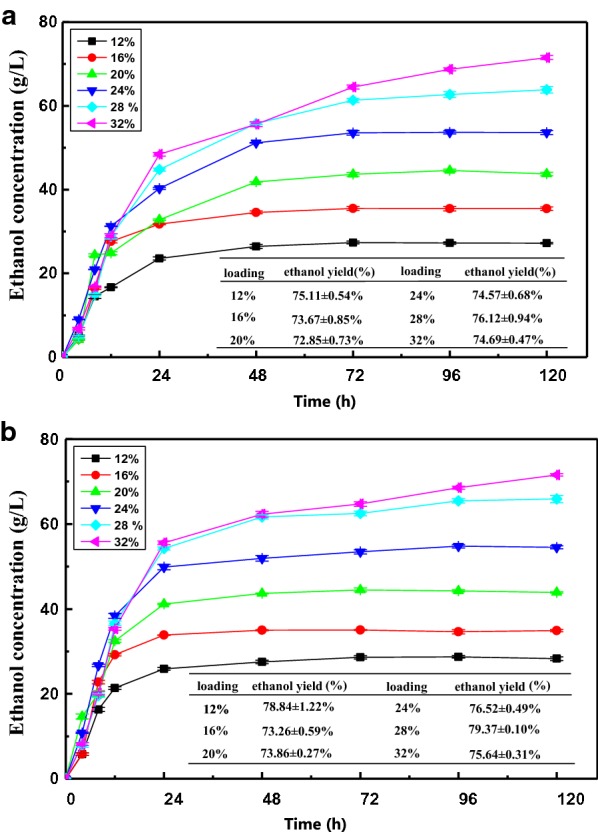
Ethanol concentrations and yields of SCB and molasses mixtures (**a** 1:3, **b** 1:1) at different solid loadings in SSF

Molasses contains a high content of fermentable sugars and other nutrients, which are beneficial to yeast growth and ethanol production [11]. Therefore, the addition of molasses to SCB ethanol production may enhance the resulting ethanol concentration. In this study, molasses was first co-fermented with pretreated SCB at a ratio of 1:3 (mixture A) with various solid loadings of 12%, 16%, 20%, 24%, 28%, and 32%, and the results are presented in Fig. 2a. With the existing of fermentable sugar in molasses, significant differences of fermentation rates in the first 12 h could be observed between the pretreated SCB alone and mixture A (*p* < 0.05). The fermentation rates of mixtures A in the first 12 h were higher (1.39–2.61 g/L/h) than those of those pretreated SCB (0.78–1.61 g/L/h). Furthermore, the ethanol concentration of mixtures A increased sharply from 0 to 24 h, and then rose steadily until it reached the highest value. When the solid loading was increased from 12 to 24%, the ethanol concentration did not present distinctive differences after 48 h. However, for substrates fermented at loadings of 28% or 32%, the ethanol concentration displayed different increased over time but increased gradually until 120 h. When the ratios of molasses and pretreated SCB were 1:3 (mixtures A), the liquidity of the system was not improved, and it remained difficult to effectively saccharify and ferment SCB at an increasing solid loadings in batch mode. To further increase the ethanol yield, more molasses was added to the pretreated SCB, with a ratio of 1:1 (mixtures B). Mixtures B had a similar variation tendency as did mixtures A in the first 24 h (Fig. 2b). In the first 12 h of SSF, there was a better fermentation rate when more molasses was added, with an ethanol productivity of 1.78–3.19 g/L/h, than was seen for mixtures A and pretreated SCB. The higher ethanol yield of mixtures B in the first 12 h of SSF can be explained by its higher fermentable sugars content, which was provided by molasses in the fermentation broth. For mixtures B, when the solid loading was increased from 12 to 24%, the ethanol concentration stabilized after 24 h; even with loadings of 28% and 32%, the rate of fermentation slowed from 48 to 72 h, indicating that most of the released fermentable sugars were converted to ethanol.

As expected, the addition of molasses can markedly improve the liquidity of the system and shorten the fermentation process. The maximum ethanol concentration of mixtures A and B was 71.54 g/L and 71.72 g/L, with ethanol yields of 74.69% and 75.64%, respectively, which were higher than those of the pretreated SCB (62.01 g/L, 64.1%). These results are attributed to the good growth environment of the mixed substrates and the high hydrolysis rate of cellulase in a relative low loading of lignocellulosic substrates [7]. It was concluded that mixture B (1:1) was optimal and worthy of further research regarding a higher solid content to further boost the ethanol concentration in batch mode, which is described in the next section.

### The co-fermentation of pretreated SCB and molasses at high solid loadings in batch mode

**Fig. 3:**
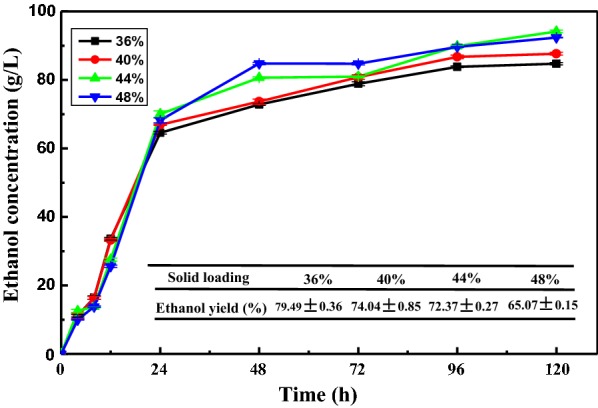
Ethanol concentrations and yields of SCB and molasses mixture (1:1) at higher gravity fermentation

The co-fermentation of pretreated SCB and molasses was carried out at a ratio of 1:1 at high solid loadings of 36%, 40%, 44%, and 48%. The results are displayed in Fig. 3. The increasing trends of ethanol concentration were nearly the same as that of mixtures A and mixtures B at 12% to 32% solid loading (Fig. 2). A rapid increase was observed in the first fermentation of 24 h, and almost the equal quantities of ethanol concentration were acquired. Thereafter, the ethanol concentration increased gradually, reaching 84.75 g/L and 87.66 g/L with solid loadings of 36% and 40%, respectively. The highest final ethanol concentration (94.20 g/L) was achieved at a solid loading of 44%, with an ethanol yield of 72.37%. At the solid loading of 48%, an ethanol concentration of only 92.37 g/L was obtained. The final ethanol yields after 120 h were 79.49%, 74.04%, 72.37%, and 65.07% for 36%, 40%, 44%, and 48%, respectively, decreasing gradually as the solid loading was increased. When the solid loading was increased to 48%, the ethanol concentration did not increase but observably decreased, with a low ethanol yield of 65.07%. This phenomenon can be attributed to the enhanced concentration of by-products and the increased viscosity of the mixed substrates, which impeded the enzymatic hydrolysis and yeast fermentation. One-way ANOVA was used to analyze the significant differences, and the results showed that the ethanol concentrations were significantly different between solid loadings of 36%, 40%, 44%, and 48% (*p* < 0.05). Similar results of high ethanol concentrations at increased solid loadings have been reported in the previous studies. Cunha et al. [35] obtained a high ethanol concentration (93.0 g/L) with a mixture of *Eucalyptus globulus* wood and cheese whey powder at high temperature and solid loadings using the industrial *Saccharomyces cerevisiae* Ethanol Red^®^ strain. Dayanand et al. obtained 83.2 g/L ethanol from birch wood at a high solid loading in gradual fed-batch feeding mode [36].

Table 2 describes the byproducts (glycerol, lactic acid, and acetic acid) of the co-fermentation of pretreated SCB and molasses at high solid loadings. Changes in the concentrations of byproducts were similar. The overall concentration trend of byproducts was shown to be increasing when higher solid loadings were used in the SSF. Glycerol is an inhibitor of SSF, because it consumes approximately 4% of the substrate carbon source in the fermentation [37]. The highest glycerol concentrations were obtained after increasing gradually as the solid loading was increased. However, glycerol first increased and then slightly decreased as the fermentation time was prolonged. The highest concentration of 7.08 g/L was obtained at 72 h, with a solid loading of 48%. The reduction of glycerol may be due to its conversion to dihydroxyacetone [38]. More lactic acid in the production would result in a decrease of the final ethanol concentration [25]. Lactic acid reached its highest value after 48 h, before falling slightly. The final concentration of lactic acid for 44% and 48% loading was quite similar, which was likely because of the lower utilization of substances by the lactic acid bacteria at high loadings. The inhibiting effect of acetic acid is related to the pH of the fermentation broth; when the pH is lowered to 4.5 and below, a lower concentration of acetic acid can reduce the final ethanol titer or even completely impede ethanol production [39]. Cantarella et al. [40] found that acetic acid concentrations of more than 2 g/L reduced the yield of ethanol fermentation when the pH of SSF was controlled at 4.8. Hanmilton et al. [41] considered that the influence of acetic acid was altogether the result of released protons and accumulated anions. The acetic acid concentration at solid loadings of 40% to 48% all exceeded 2 g/L, indicating that higher solid loadings had a negative effect on ethanol yield. The concentrations of glycerol, lactic acid, and acetic acid were all increased upon increasing solid loadings, which lead to lower ethanol production. To reduce the cost of distillation and minimize the effects of by-products caused by a high solids system, a solid loading of 36% may be a better choice for the co-fermentation of pretreated SCB and molasses, with a ratio of 1:1, to obtain higher ethanol titers.

**Table 2:** Byproducts (glycerol, lactic acid, and acetic acid) of co-fermentation of SCB and molasses at higher loadings

	Loading (%)	Concentrations of byproducts			
		12 h	48 h	72 h	120 h
Glycerol	36	4.25 ± 0.15	5.62 ± 0.26	5.38 ± 1.01	5.19 ± 0.23
	40	4.54 ± 0.09	6.14 ± 0.76	5.86 ± 0.36	5.22 ± 0.10
	44	4.06 ± 0.79	6.48 ± 0.33	6.2 ± 0.10	6.02 ± 0.78
	48	4.04 ± 0.96	7.08 ± 0.13	6.63 ± 0.71	6.49 ± 1.00
Lactic acid	36	4.15 ± 0.26	5.25 ± 0.54	3.84 ± 0.39	3.03 ± 0.26
	40	4.8 ± 0.53	4.48 ± 0.46	4.46 ± 0.19	3.99 ± 0.13
	44	5.17 ± 0.03	5.31 ± 0.95	4.68 ± 0.57	4.61 ± 0.15
	48	5.11 ± 0.39	5.67 ± 0.63	5.16 ± 0.44	4.62 ± 0.55
Acetic acid	36	1.99 ± 0.20	2.02 ± 0.10	2.10 ± 0.50	1.91 ± 0.19
	40	2.01 ± 0.05	2.05 ± 0.06	2.12 ± 0.04	2.12 ± 0.55
	44	2.04 ± 0.13	2.02 ± 0.11	2.1 ± 0.13	2.12 ± 0.43
	48	2.03 ± 0.30	2.12 ± 0.30	2.12 ± 0.21	2.22 ± 0.29

### Anaerobic digestion of the residual stillage to produce biogas

**Fig. 4:**
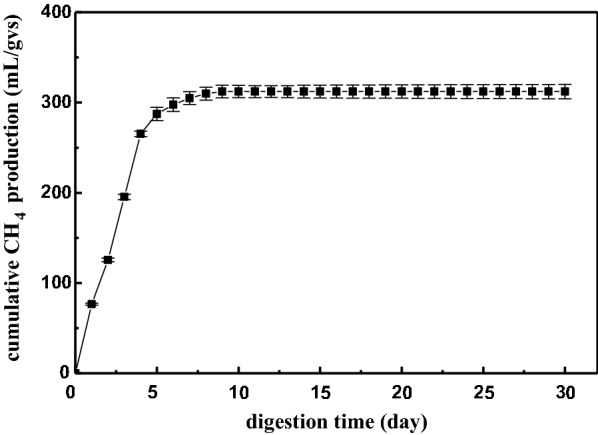
Total methane production by anaerobic digestion after SSF

After SSF, the ethanol was removed by evaporation, and the residual stillage of the batch system (36% w/w) was subjected to methane production by anaerobic digestion. Figure 4 illustrates the accumulated methane production in mL/gVS under the standard temperature and pressure conditions. In general, there are four degradation stages during anaerobic digestion: hydrolysis, acidogenesis, acetogenesis or dehydrogenation, and methanation [42]. However, the fermentation residue mainly contained non-fermentable sugars, such as pentose, which cannot be used by general yeast strains, as well as fermentation byproducts (glycerol, lactic acid, and acetic acid). The rate of methane production was faster in the first 4 days, with a value of 66.31 mL/g VS per day. After this time, it rose modestly until halting. The final VS degradation efficiency of 95.93% indicated that the inoculum was easily adapted to the fermentation substrates, as discussed above. A final methane yield of 312.14 mL/g VS was obtained after 30 days, which was in accordance with the previous reports. Liu et al. [43] evaluated sequential bioethanol and biogas production based on high solids’ SSF and obtained 306.97 mL/gVS methane from alkali-pretreated SCB. The result achieved in this thesis was likely due to the high organic content in molasses and mixed substrate characteristics. After anaerobic digestion, the COD removal efficiency was 85.9%, with an initial concentration of 13,500 mg/L. Several similar studies have reported COD removal of 70–95% from the biogas production of various stillage types [44]. The high COD removal values resulted in high methane production, and it was concluded that most of the organic substrates were effectively converted to biogas.

Based on the heating values of 30.0 kJ/g (ethanol) and 35.9 kJ/L (methane), the energy values achieved from the co-production of ethanol and biomethane were 253.2 kJ and 128.5 kJ, respectively, which were higher than when producing only bioethanol. Dayanand et al. [36] observed a higher yield in the ethanol and biogas production from birch wood than when producing either ethanol or biogas alone. Similarly, Cavka et al. [45] demonstrated that the co-production of biofuels from hemp stem was more than twice the energy values of ethanol production alone. Mass balance is necessary to evaluate the overall process of biofuel production from biomass. A total of 1800 g of pretreated SCB and 1800 g of molasses were mixed, and the sequential bioethanol and biogas production was calculated. The calculated ethanol and biogas generation was 847 g and 596.6 L, respectively. Biofuels are more sustainable and environmental friendly than fossil fuels. They can reduce greenhouse gas emissions and help to relieve the energy crisis. Based on this viewpoint, distilled stillage was more favorable for anaerobic digestion.

## Conclusions

Integrating sugarcane molasses into SCB ethanol production not only increased the final ethanol concentration but also obtained a suitable conversion rate. Compared to pretreated SCB fermentation alone, the addition of molasses showed greater advantages by improving the liquidity of the system, shortening the fermentation time, and yielding higher ethanol production. At a mixture ratio of 1:1, the highest ethanol concentration of 94.20 g/L was obtained at a solid loading of 44%, with a fermentation yield of 72.37%. After distillation, biogas production from the residual stillage was conducted by anaerobic digestion, yielding 312.14 mL methane/g VS within 30 days, resulting in the removal of 85.9% COD. Thus, the sequential production of bioethanol and biogas allows us to achieve a comprehensive utilization of biomass resources with associated environmental benefits.

## Methods

### Materials

The SCB and molasses used in this study were collected in the month of December from Maoyuan Sugar Co., Ltd., in Shaoguan, China. SCB was first milled into < 1 mm lengths with a MiniMill (MF10, IKA, Germany), sealed in a sealing bag, and stored at room temperature. The molasses sample was kept at 4 °C in a refrigerator for further utilization. The chemical compositions of untreated and pretreated SCB were determined using the method provided by the NREL [46]. The fermentation sugars in molasses were quantified by a high-performance liquid chromatography system (HPLC, Shimadzu, Japan) equipped with a refractive index detector (RID) and a cation-exchange column (SUGAR KS-801; 300 mm * 8.0 mm; Shodex™, Japan). The compositions of SCB and molasses are shown in Table 1.

Cellulase (Cellic CTec2) was obtained from Novozymes (Bagswade, Denmark), with an enzyme activity of 164 FPU/mL according to the methods of NREL [47].

The microorganism strain *Saccharomyces cerevisiae* was provided by Angel Yeast Co., Ltd. (Yichang, China).

### Alkaline pretreatment

Alkali pretreatment of SCB was performed in a round-bottom flask. An aliquot of 50 g SCB (dry basis) was added to 1 L of 0.5 M NaOH and incubated at 80 °C for 2 h with 150 rpm. At the end of the pretreatment, the liquor and solid residues were separated by filtration. The slurry was washed with water until the washing liquid had a neutral pH. The obtained residues were dried at 50 °C for 48 h, and stored for composition analysis and subsequent fermentation processing.

### Simultaneous saccharification and fermentation (SSF)

Before SSF, the dry yeast (6.6 g) was activated in a 2% glucose solution (100 mL) in two steps: 36 °C for 10 min and 34 °C for 60 min in a shaker at 120 rpm. The SSF process was used in this research to convert biomass into ethanol. The alkali-pretreated SCB and molasses at various ratios was added into a 250 mL flask. The pH of the fermentation system was adjusted to 4.5 using 1.0 mol/L H_2_SO_4_. After autoclaving at 121 °C for 20 min, certain amounts of cellulase (15 FPU/g pretreated SCB) and activated yeast (5 mL) were added into the system [6]. A specified volume of sterile water was then added to the mixture to reach 100 mL of liquor. The fermentation was performed at 34 °C and 120 rpm for 120 h in a shaker. SSF trials were conducted in triplicate, and the results are reported as mean values.

During SSF, the quantities of sugars and ethanol collected at different times were determined by HPLC using a refractive index detector (RID) and a cation-exchange column (SUGAR SH1011; 300 mm * 8.0 mm; Shodex™, Japan). 0.05 M H_2_SO_4_ was used as the mobile phase with a flow rate of 1 mL/min at column temperature of 50 °C. The byproducts (glycerol, lactic acid, and acetic acid) were separated using an HPLC system equipped with a Prominence UV/Vis detector (SPD) and an ion-exchange column (SUPELCOGEL C-610H; 300 mm * 7.8 mm; Shodex™, Japan). In theory, 1 g of cellulose can be hydrolyzed into 1.11 g of glucose and that 1 g of glucose can be converted into 0.511 g of ethanol during the SSF process [48]. The ethanol yield was calculated based on the following equation:

$${\text{Ethanol yield (\% )}} = \frac{\text{Actual ethanol released}}{\text{Theoretical ethanol release}} \times 100\% .$$

### Biogas production

Ethanol from the fermentation medium was collected using a rotary evaporator at 60 °C for 30 min under reduced pressure. The residues in the stillage were used in the subsequent biogas production experiments. Biological methane potential (BMP) detection was conducted in triplicate using the Bioprocess Control AMPTS II (automatic methane potential test system). The experiments were performed in 500 mL sealed batch flasks with a working volume of 400 mL at 37 °C for 30 days, in which the mixed ratio of substrates and inoculum was 1:1 based on the VS amount in grams.

The inoculum, obtained from our laboratory, was trained chronically in a mesophilic anaerobic tank with total solids (TS) 6.74%, volatile solids (VS) 1.18%, and a pH of 7.3–7.5. Before starting the assay tests, the inoculum was incubated in a starving condition at 37 °C for 1 week to lessen the endogenous biogas production. Pure N_2_ was introduced into the headspace of the bottle for 3–5 min to ensure anaerobic conditions. A blank sample consisting of pure inoculum and a contrast sample consisting of pure microcrystalline cellulose were used as controls. The experiments were carried out until no gas was detected.

Samples were withdrawn at different times for determining the constituent elements. During the biogas production process, the determinations of TS, VS, COD, and ammonium nitrogen (NH_4_-N) were based on the methods provided by a previous report [49], and the pH was measured using a pH meter (Five Easy Plus, Mettler-Toledo, Australia).

### Statistical analysis

Analysis of standard errors and variance was performed using SPSS version 16.0. Statistical differences in the ethanol concentrations among the different solid loadings and in the fermentation rates between pure SCB and the mixtures were analyzed using one-way ANOVA. ‘‘significant” was used when the *p* value < 0.05.

## Abbreviations

SCB: sugarcane bagasse
VS: volatile solids
COD: chemical oxygen demand
SSF: simultaneous saccharification and fermentation
SHF: the separate hydrolysis and fermentation
HPLC: high-performance liquid chromatography
RID: refractive index detector
BMP: biological methane potential
AMPTS: automatic methane potential test system
TS: total solid
VS: volatile solid
